# Shadow Filters Using Multiple-Input Differential Difference Transconductance Amplifiers

**DOI:** 10.3390/s23031526

**Published:** 2023-01-30

**Authors:** Montree Kumngern, Fabian Khateb, Tomasz Kulej

**Affiliations:** 1 Department of Telecommunications Engineering, School of Engineering, King Mongkut’s Institute of Technology Ladkrabang, Bangkok 10520, Thailand; 2 Department of Microelectronics, Brno University of Technology, Technická 10, 601 90 Brno, Czech Republic; 3 Faculty of Biomedical Engineering, Czech Technical University in Prague, nám. Sítná 3105, 272 01 Kladno, Czech Republic; 4 Department of Electrical Engineering, University of Defence, Kounicova 65, 662 10 Brno, Czech Republic; 5 Department of Electrical Engineering, Czestochowa University of Technology, 42-201 Czestochowa, Poland

**Keywords:** shadow filter, differential difference transconductance amplifier, multiple-input MOS technique, analog filter

## Abstract

This paper presents new voltage-mode shadow filters employing a low-power multiple-input differential difference transconductance amplifier (MI-DDTA). This device provides multiple-input voltage-mode arithmetic operation capability, electronic tuning ability, high-input and low-output impedances. Therefore, the proposed shadow filters offer circuit simplicity, minimum number of active and passive elements, electronic control of the natural frequency and the quality factor, and high-input and low-output impedances. The proposed MI-DDTA can work with supply voltage of ±0.5 V and consumes 9.94 μW of power. The MI-DDTA and shadow filters have been designed and simulated with the SPICE program using 0.18 μm CMOS process parameters to validate the functionality and workability of the new circuits.

## 1. Introduction

The universal filters are the systems that can realize several filtering functions into the same topology such as low-pass (LP), high-pass (HP), band-pass (BP), band-stop (BS), and all-pass (AP) filters, usually with second-order transfer functions [1,2,3,4,5]. These second-order filters can be applied for three-way high-fidelity loud-speakers, phase-locked loops, and high-order filters [6,7,8]. The filters with orthogonal control of the natural frequency and the quality factor are usually required because it is easy to design the required operating frequency and the required quality factor.

The shadow filter was first introduced in [9]. It consists of a conventional universal filter with LP and BP outputs, summing circuit, and an external amplifier. The output signal of the LP filter is amplified by the external amplifier and fed back to the summing circuit at the input of the universal filter. The adjustable gain of the amplifier can be used to modify the natural frequency and the quality factor of the universal filters, which is valuable for trimming the parameters of filters when non-ideal effects are occurred. The concept of the shadow filter in [9] was developed next to obtain both modification of the natural frequency and the quality factor with an external amplifier [10].

There are many shadow filters (also known as frequency-agile filters) realized using variant active elements available in the literature [11,12,13,14,15,16,17,18,19,20]. In [11,12,13,14,15,16,17,18], current-mode (CM) shadow filters have been reported whereas in [19,20,21,22,23,24,25,26,27,28,29,30] voltage-mode (VM) shadow filters have been introduced. This paper is focused on the VM filters which offer high-input and low-output impedances, electronic tuning ability, and use grounded passive components. The VM shadow filters using active elements such as operational trans-resistance amplifier (OTRA) [19], current-feedback operational amplifier (CFOA) [20,21], and differential-difference current conveyor (DDCC) [22] have been previously introduced in literature. However, the circuits in [19,20,21,22] don’t provide an electronic tuning ability of the natural frequency and the quality factor. The shadow filters employing voltage differencing transconductance amplifier (VDTA) [23,24,25,26,27], voltage differencing gain amplifier (VDGA) [28], voltage differencing differential difference amplifier (VDDDA) [29], and operational transconductance amplifier (OTA) [30] offer an electronic tuning ability and high-input impedance, which is advantageous for VM circuits. However, these active filters were supplied with relatively high voltages, namely, ±0.9 V in [23,29], ±1 V in [24,25,27], ±1.5 V in [26], and ±1.8 V in [30].

In this paper a new voltage-mode shadow filters using low-voltage and low-power multiple-input differential difference transconductance amplifiers (MI-DDTA) have been proposed. The MI-DDTA offers multiple-input addition and subtraction of voltages, which is possible by using the multiple-input gate-driven MOS transistor (MIGD MOST) technique [31,32,33,34,35,36]. The proposed filters offer high-input and low output impedance which is required for cascading in voltage-mode circuits. The natural frequency and the quality factor can be controlled electronically. The proposed circuits can work with ±0.5 V supply and they have been designed and simulated with SPICE, using 0.18 μm CMOS process parameters to verify the functionality and workability of the new circuits.

It is worth noting that the DDTAs using multiple-input bulk-driven MOST technique have been proposed already in [37,38,39]. The DDTAs in [37,38] use a 0.5 V of supply voltage, and the DDTA in [39] uses a 0.3 V of supply voltage. These DDTAs consume ultra-low power in the range of nano watt; however, they are suitable for applications operating with limited bandwidth in the range of a few hundred Hz like applications in biomedical systems.

## 2. Proposed Circuit

### 2.1. Proposed MI-DDTA

**Figure 1:**
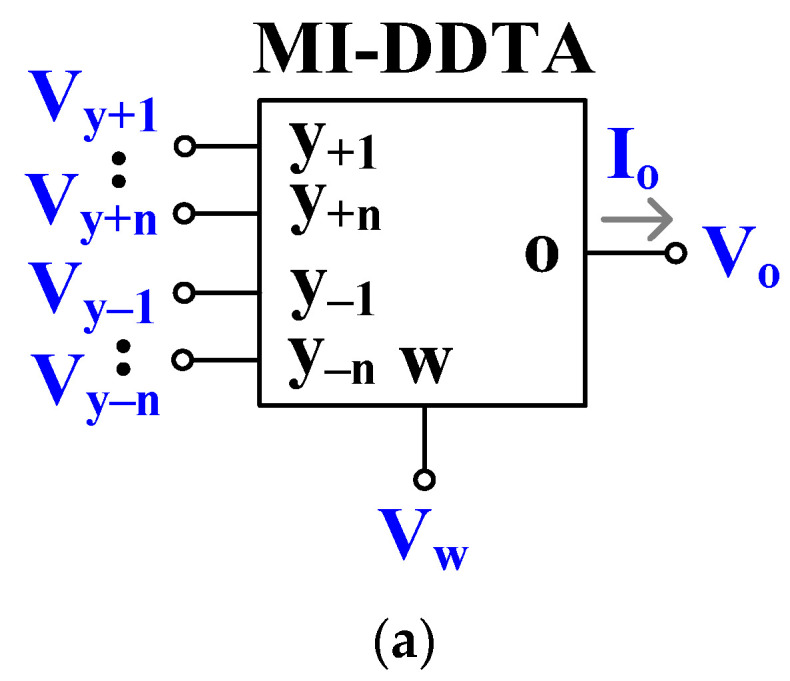

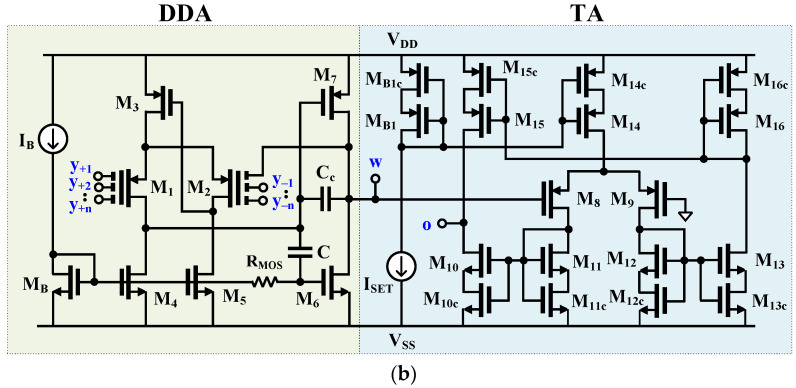
Proposed MI-DDTA: (**a**) the electrical symbol and (**b**) possible CMOS implementation.

Figure 1a shows the electrical symbol of the MI-DDTA. The low-frequency characteristics of the device are given by:

(1) $$\left.\begin{array}{c} V_{w}=V_{y+1}+V_{y+2}-V_{y-1}-V_{y-2}\\ I_{o}=g_{m}V_{w} \end{array}\right\}$$

In brief, the operation of the circuit could be characterized as follows: the voltage at the low-impedance output *w* is a sum of two differential input voltages $V_{y1}=V_{y+1}-V_{y-2}$ and $V_{y2}=V_{y+2}-V_{y-1}$. The output current $I_{o}$ at the high-impedance output *o* is equal to the product of $V_{w}$, and a transconductance gain $g_{m}$, namely, the second equation of (1) describes a voltage-controlled current source. The CMOS structure of the proposed MI-DDTA is shown in Figure 1b. It consists of a differential difference amplifier (DDA) with unity gain feedback, followed by a transconductance amplifier (TA).

The unity gain DDA consists of a differential stage based on the flipped voltage follower M_1_-M_5_ that allowed the minimum voltage supply to be as low as the sum of one gate-source (*V_GS-M3_*) and one drain source (*V_DS-M5_*) voltage, i.e., *V_DDmin_ = V_GS-M3_ + V_DS-M5_*; hence, the low voltage supply capability is guaranteed. To increase the number of inputs of the differential pair M_1_ and M_2_, the multiple-input gate-driven MOS transistor (MI-GD-MOST) technique is used [31,32,33,34,35,36]. This multiple-input increase the arithmetic operation capability of the DDTA circuit.

**Figure 2:**
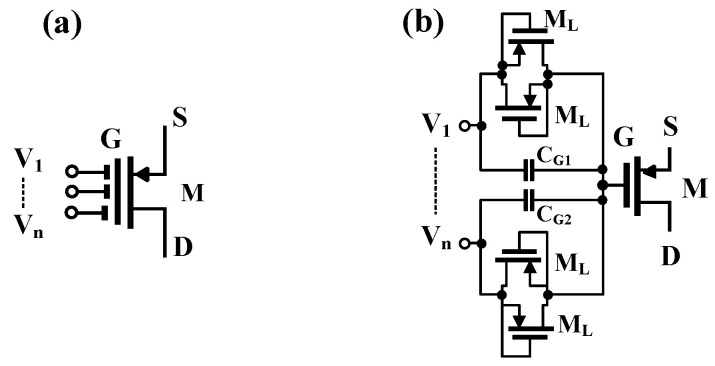
MIGD-MOST: (**a**) symbol and (**b**) implementation.

The symbol of the MI-GD-MOST and its implementation is shown in Figure 2. The arbitrary number of inputs *V_1_*, … *V_n_* are simply obtained by parallel connection of the input capacitor C_G_ and two anti-parallel MOS transistors M_L_ operating in cutoff region, hence creating high resistance value with minimum occupied chip area. These high resistances are essential for proper DC operation of the circuit while the input capacitors ensure the AC path for the input signals. The second stage of the unity gain DDA is created by class-AB stage M_6_, M_7_ and high resistance R_MOS_. This R_MOS_ is also created by two cut-off transistors M_L_, and ensures the proper DC bias current of this output stage, while the capacitor C ensures the AC path for the signal; hence a simple class-AB stage is obtained. The compensation capacitor C_c_ ensure the stability of the DDA circuit.

(2) $$\beta =\frac{\alpha \cdot g_{m1,2}\left(r_{ds1,2}||r_{ds4,5}\right)\left(g_{m6}+g_{m7}\right)\left(r_{ds1,2}||r_{ds4,5}\right)}{1+\alpha \cdot g_{m1,2}\left(r_{ds1,2}||r_{ds4,5}\right)\left(g_{m6}+g_{m7}\right)\left(r_{ds1,2}||r_{ds4,5}\right)}$$

where *β* is the voltage gain of the capacitive voltage divider, at the gates of M_1_ and M_2_, which neglecting the impact of the parasitic capacitances of MOS transistor, can be approximated as:

(3) $$\alpha ≅\frac{C_{G1}}{\sum_{i=0}^{n}C_{Gi}}$$

where *n* is the number of differential inputs of the MI- DDTA (note that one more capacitor *C_G0_* is used in feedback connection). Assuming *n* = 2 and *C_G0_ = C_G1_ = C_G2_* results in α = 1/3. The output resistance seen at the *w* terminal, *R_w_*, is given by:

(4) $$R_{w}≅\frac{1}{\alpha \cdot g_{m1,2}\left(r_{ds1,2}||r_{ds4,5}\right)\left(g_{m6}+g_{m7}\right)}$$

Note, that both the low-frequency gain *β*, as well as the output resistance *R_w_*, are deteriorated by the input capacitive divider. However, thanks to the two-stage architecture of the internal OTA, used to create the MI-DDTA, and class AB operation, both parameters can achieve acceptable values.

The input capacitance seen from a single *y* terminal, for identical capacitances *C_G_*_,_ which are much larger than parasitic capacitances of an MOS transistor, is:

(5) $$C_{y}≅\frac{2}{3}C_{G}$$

The gain bandwidth product (GBW) of the internal OTA, M_1_–M_7_, which is approximately equal to the 3-dB frequency of the gain *β*, depends on the transconductance of the input differential stage and the compensation capacitor *C_C_*:

(6) $$GBW=\frac{\alpha \cdot g_{m1,2}}{C_{c}}$$

The transconductance stage is realized using the mirror topology M_8_–M_16_. The structure employs the self-cascode connections M_10c_–M_16c_ in order to increase the output resistance and the gain of the TA. The current I_SET_ can be used to regulate the transconductance of the TA, which in the weak inversion region, with unity-gain current mirrors, is given by:

(7) $$g_{m}=\frac{I_{SET}}{n_{p}U_{T}}$$

where *n_p_* is the subthreshold slope factor for a p-channel MOS transistor and *U_T_* is the thermal potential.

The output resistance of the TA, i.e., the resistance seen from its *o* output, *R_o_*, can be approximated as:

(8) $$R_{o}≅(g_{m10}r_{ds10}r_{ds10c})||(g_{m15}r_{ds15}r_{ds15c})$$

and its DC voltage gain *A_TA_* is given by:

(9) $$A_{TA}=g_{m}R_{o}$$

The parasitic poles associated with internal nodes of the TA are located well above the GBW product of the TA, consequently, the GBW product depends on the loading capacitance at the *o* terminal C_LTA_, and is given by:

(10) $$GBW_{TA}=\frac{g_{m}}{C_{LTA}}$$

### 2.2. Proposed Shadow Filters

**Figure 3:**
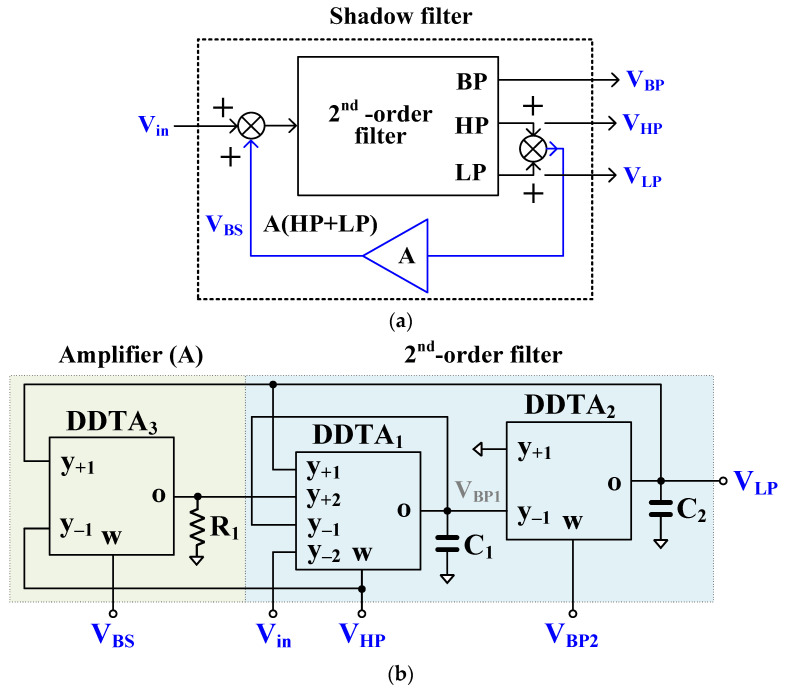
First proposed shadow filter: (**a**) block diagram of the first shadow filter [10], (**b**) proposed first shadow filter using DDTAs.

Figure 3a shows the block diagram of the shadow filter [10] that has been used to realize the first proposed shadow filter as shown in Figure 3b. The DDTA1, DDTA2 along with capacitors *C1* and *C2* realize the 2nd-order filter while DDTA3 along with resistor R1 realize the amplifier (A). The inverting high-pass (HP) and band-pass (BP) responses are obtained at the VHP and VBP1 outputs, respectively, while VLP, VBP2, and VBS provide non-inverting low-pass (LP), BP, and band-stop (BS) responses. It should be noted that the input Vin possesses high impedance and outputs VHP, VBP2, and VBS possess low impedance while the output VLP needs the buffer circuit if low-impedance load is connected. The outputs VLP and VHP are summed and amplified. Using (1) and nodal analysis, the transfer functions of LP, HP, BP and BS filters can be expressed by:

(11) $$\frac{V_{LP}}{V_{in}}=\frac{g_{m1}g_{m2}\left(\frac{1}{1+g_{m3}R_{1}}\right)}{s^{2}C_{1}C_{2}+sC_{2}g_{m1}\left(\frac{1}{1+g_{m3}R_{1}}\right)+g_{m1}g_{m2}}$$

(12) $$\frac{-V_{HP}}{V_{in}}=\frac{s^{2}C_{1}C_{2}\left(\frac{1}{1+g_{m3}R_{1}}\right)}{s^{2}C_{1}C_{2}+sC_{2}g_{m1}\left(\frac{1}{1+g_{m3}R_{1}}\right)+g_{m1}g_{m2}}$$

(13) $$\frac{V_{BP2}}{V_{in}}=\frac{-V_{BP1}}{V_{in}}=\frac{sC_{2}g_{m1}\left(\frac{1}{1+g_{m3}R_{1}}\right)}{s^{2}C_{1}C_{2}+sC_{2}g_{m1}\left(\frac{1}{1+g_{m3}R_{1}}\right)+g_{m1}g_{m2}}$$

(14) $$\frac{V_{BS}}{V_{in}}=\frac{s^{2}C_{1}C_{2}\left(\frac{1}{1+g_{m3}R_{1}}\right)+g_{m1}g_{m2}\left(\frac{1}{1+g_{m3}R_{1}}\right)}{s^{2}C_{1}C_{2}+sC_{2}g_{m1}\left(\frac{1}{1+g_{m3}R_{1}}\right)+g_{m1}g_{m2}}$$

where $g_{m3}R_{1}=A$. The gain A can be regulated by $g_{m3},$ with constant $R_{1}$, or by regulating $R_{1}$ with constant $g_{m3}$.

The natural frequency ($\omega_{o}$) and the quality factor (Q) are given by:

(15) $$\omega_{o}=\sqrt{\frac{g_{m1}g_{m2}}{C_{1}C_{2}}}$$

(16) $$Q=\left(1+A\right)\sqrt{\frac{g_{m2}C_{1}}{g_{m1}C_{2}}}$$

From (15), the parameter $\omega_{o}$ can be controlled by $g_{m1}\;$=$\;g_{m2}$ and from (16), the parameter Q can be controlled by A (i.e., regulating g_m3_ with constant $R_{1}$) while maintaining $g_{m1}$ = $g_{m2}$ and $C_{1}$ = $C_{2}$. Thus, the parameters $\omega_{o}$ and Q can be controlled electronically.

From (11)–(14), increasing the parameter Q will decrease the passband of LP and HP filters by $1/\left(1+A\right)$, whereas the gain of the BP filter will be constant.

It should be noted from (16) that the parameter Q can be increased if A>1. When the amplifier inputs are swapped (i.e., connecting the y_−1_-terminal to V_LP_ and the y_+1_-terminal to V_HP_), the parameter Q will be proportional to $\left(1-A\right)$. In this case 0<A<1 is used.

**Figure 4:**
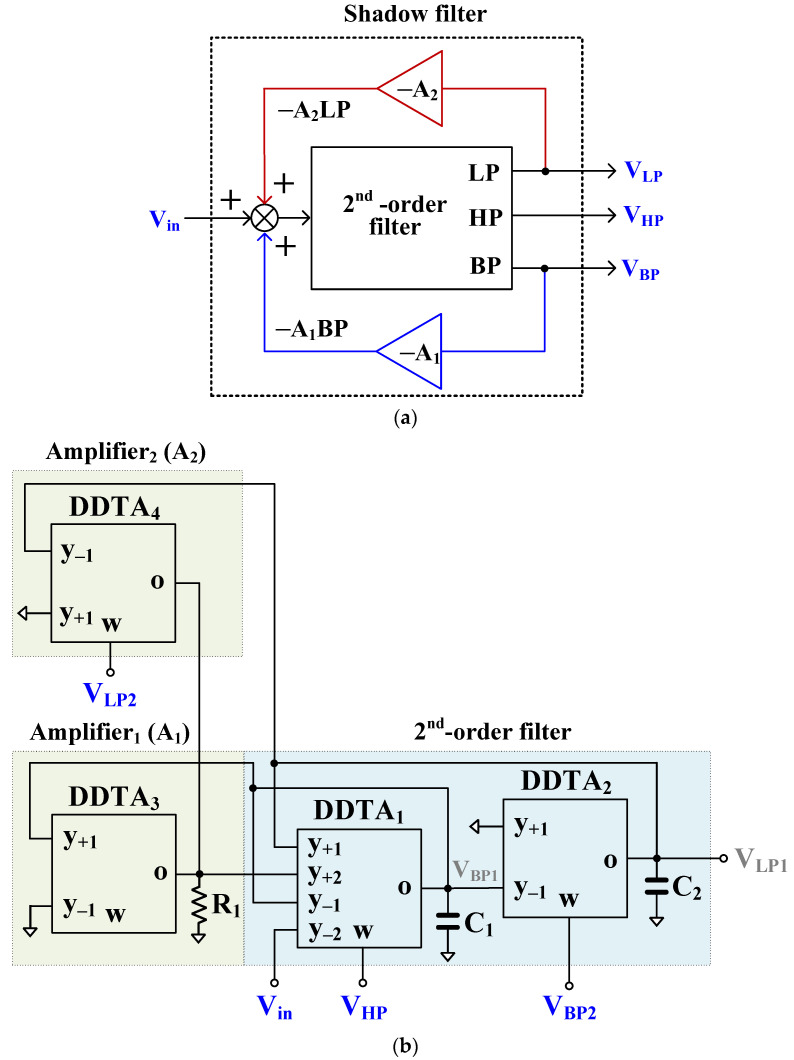
Second proposed shadow filter: (**a**) block diagram of the second shadow filter [10], (**b**) proposed second shadow filter using DDTAs.

The block diagram of the second proposed shadow filter is shown in Figure 4a. In this system two amplifiers A_1_ and A_2_ are used to amplify the output signals V_BP_ and V_LP_, respectively. The proposed filter, employing four MI-DDTAs, two grounded capacitors, and one resistor is shown in Figure 4b. The DDTA_1_ and DDTA_2_, along with capacitors *C_1_* and *C_2_* are used to realize the 2nd order filter while the resistor R_1_ along with the DDTA_1_ and DDTA_2_, respectively, are used to realize the amplifiers A_1_ and A_2_. The non-inverting LP and BP responses are obtained at the V_LP1_ and V_BP2_ outputs, respectively, while V_LP2_, V_HP_, and V_BP1_ provide inverting LP, HP, and BP responses. It should be noted that the input V_in_ possesses high impedance while the outputs V_LP2_, V_HP_, and V_BP2_ possess low impedance. The output V_BP1_ is amplified by A_1_ using DDTA_3_ and resistor *R_1_* and the output V_LP1_ is amplified by A_2_ using DDTA_4_ and the same resistor *R_1_*.

Using (1) and nodal analysis, the transfer function of the second proposed filter in Figure 4b can be expressed by:

(17) $$\frac{V_{LP1}}{V_{in}}=\frac{-V_{LP2}}{V_{in}}=\frac{g_{m1}g_{m2}}{s^{2}C_{1}C_{2}+sC_{2}g_{m1}\left(1-g_{m3}R_{1}\right)+g_{m1}g_{m2}\left(1-g_{m4}R_{1}\right)}$$

(18) $$\frac{-V_{HP}}{V_{in}}=\frac{s^{2}C_{1}C_{2}}{s^{2}C_{1}C_{2}+sC_{2}g_{m1}\left(1-g_{m3}R_{1}\right)+g_{m1}g_{m2}\left(1-g_{m4}R_{1}\right)}$$

(19) $$\frac{V_{BP2}}{V_{in}}=\frac{-V_{BP1}}{V_{in}}=\frac{sC_{2}g_{m1}}{s^{2}C_{1}C_{2}+sC_{2}g_{m1}\left(1-g_{m3}R_{1}\right)+g_{m1}g_{m2}\left(1-g_{m4}R_{1}\right)}$$

where $g_{m3}R_{1}=A_{1}$ and $g_{m4}R_{1}=A_{2}$. From (17)–(19), they are valid for $A_{1}<1$ and $A_{2}<1$. The natural frequency of the filter and its quality factor can be expressed as:

(20) $$\omega_{o}=\sqrt{1-A_{2}}\sqrt{\frac{g_{m1}g_{m2}}{C_{1}C_{2}}}$$

(21) $$=\frac{\sqrt{1-A_{2}}}{1-A_{1}}\sqrt{\frac{g_{m2}C_{1}}{g_{m1}C_{2}}}$$

From (20), the parameter $\omega_{o}$ can be controlled by $A_{2}$ through adjusting $g_{m4}$ or by adjusting $g_{m1}=g_{m2}$. However, adjusting the parameter $\omega_{o}$ by $A_{2}$ affects the parameter Q. The parameter Q can be controlled by $A_{1}$ without affecting the parameter $\omega_{o}$ through adjusting $g_{m3}$. From (17)–(20), adjusting the parameter $\omega_{o}$ will change the passband of LP and BP filters whereas the passband of HP filter is constant.

### 2.3. Non-Idealities Analysis

The non-idealities of DDTA can be considered as

(22) $$\left.\begin{array}{c} V_{w}=\beta_{+j1}V_{y+1}+\beta_{+j2}V_{y+2}-\beta_{-j1}V_{y-1}-\beta_{-j2}V_{y-2}\\ I_{o}=g_{mnj}V_{w} \end{array}\right\}$$

where $\beta_{+j1}=1-\varepsilon_{+j1v}$ and $\varepsilon_{+j1v}(\left|\varepsilon_{+j1v}\right|\ll 1$) denote the voltage tracking error from $V_{y+1}$ to $V_{w}$ of the j-th DDTA, $\beta_{+j2}=1-\varepsilon_{+j2v}$ and $\varepsilon_{+j2v}(\left|\varepsilon_{+j2v}\right|\ll 1$) denote the voltage tracking error from $V_{y+2}$ to $V_{w}$ of the j-th DDTA,$\;\beta_{-j1}=1-\varepsilon_{-j1v}$ and $\varepsilon_{-j1v}\;(\left|\varepsilon_{-j1v}\right|\ll 1$) denote the voltage tracking error from $V_{y-1}$ to $V_{w}$ of the j-th DDTA, and $\;\beta_{-j2}=1-\varepsilon_{-j2v}$ and $\varepsilon_{-j2v\;}(\left|\varepsilon_{-j2v}\right|\ll 1$) denote the voltage tracking error from $V_{y-1}$ to $V_{w}$ of the j-th DDTA, and $g_{mnj}$ is the frequency-dependent transconductance that typically determined the operation frequency $\omega_{o}$ [40]. The non-ideal of transconductance $g_{mnj}$ can be expressed as [41,42].

(23) $$g_{mnj}\left(s\right)≅g_{mj}\left(1-\mu_{j}s\right)$$

The non-ideal first transfer function of the proposed shadow filter in Figure 3a can be expressed by:

(24) $$\frac{V_{LP}}{V_{in}}=\frac{g_{mn1}g_{mn2}\beta_{-22}\beta_{-32}\left(\frac{1}{1+g_{m3}R_{1}\beta_{-11}\beta_{+22}}\right)}{\begin{array}{c} s^{2}C_{1}C_{2}+sC_{2}g_{mn1}\beta_{-21}\left(\frac{1}{1+g_{m3}R_{1}\beta_{-11}\beta_{+22}}\right)\\ +g_{mn1}g_{mn2}\beta_{-31}\left(\frac{\beta_{+21}+g_{m3}R_{1}\beta_{+11}\beta_{+22}}{1+g_{m3}R_{1}\beta_{-11}\beta_{+22}}\right) \end{array}}$$

(25) $$\frac{-V_{HP}}{V_{in}}=\frac{s^{2}C_{1}C_{2}\beta_{-22}\left(\frac{1}{1+g_{m3}R_{1}\beta_{-11}\beta_{+22}}\right)}{\begin{array}{c} s^{2}C_{1}C_{2}+sC_{2}g_{mn1}\beta_{-21}\left(\frac{1}{1+g_{m3}R_{1}\beta_{-11}\beta_{+22}}\right)\\ +g_{mn1}g_{mn2}\beta_{-31}\left(\frac{\beta_{+21}+g_{m3}R_{1}\beta_{+11}\beta_{+22}}{1+g_{m3}R_{1}\beta_{-11}\beta_{+22}}\right) \end{array}}$$

(26) $$\frac{V_{BP2}}{V_{in}}=\frac{-V_{BP1}}{V_{in}}=\frac{sC_{2}g_{mn1}\beta_{-22}\left(\frac{1}{1+g_{m3}R_{1}\beta_{-11}\beta_{+22}}\right)}{\begin{array}{c} s^{2}C_{1}C_{2}+sC_{2}g_{mn1}\beta_{-21}\left(\frac{1}{1+g_{m3}R_{1}\beta_{-11}\beta_{+22}}\right)\\ +g_{mn1}g_{mn2}\beta_{-31}\left(\frac{\beta_{+21}+g_{m3}R_{1}\beta_{+11}\beta_{+22}}{1+g_{m3}R_{1}\beta_{-11}\beta_{+22}}\right) \end{array}}$$

where $g_{mn3}R_{1}=A$

(27) $$\omega_{o}=\sqrt{\frac{g_{mn1}g_{mn2\beta_{-31}}}{C_{1}C_{2}}\left(\frac{\beta_{21+}+g_{mn3}R_{1}\beta_{+11}\beta_{22+}}{1+g_{mn3}R_{1}\beta_{-11}\beta_{22+}}\right)}$$

Using (23), $D\left(s\right)$ of the transfer functions can be rewritten as:

(28) $$Q=\frac{\left(1+g_{mn3}R_{1}\beta_{-11}\beta_{+22}\right)}{\beta_{-21}}\sqrt{\frac{C_{1}g_{mn2}\beta_{-31}}{C_{2}g_{mn1}}\left(\frac{\beta_{21+}+g_{mn3}R_{1}\beta_{+11}\beta_{22+}}{1+g_{mn3}R_{1}\beta_{-11}\beta_{22+}}\right)}$$

where:

(29) $$\begin{array}{l} s^{2}C_{1}C_{2}\left(1-\frac{C_{2}g_{m1}\mu_{1}\beta_{-21}\left(B\right)-g_{m1}g_{m2}\mu_{1}\mu_{2}\beta_{-31}\left(C\right)}{C_{1}C_{2}}\right)\\ \;\;\;\;\;\;+sC_{2}g_{m1}\beta_{-21}\left(B\right)(1\\ \;\;\;\;\;\;-\frac{\left(g_{m1}g_{m2}\beta_{-31}\mu_{1}+g_{m1}g_{m2}\beta_{-31}\mu_{2}\right)\left(C\right)}{C_{2}g_{m1}\beta_{-21}\left(B\right)})+g_{m1}g_{m2}\beta_{-31}\left(C\right) \end{array}$$

where:

$$B=\frac{1}{1+g_{m3}R_{1}\beta_{-11}\beta_{+22}},\;C=\frac{\beta_{+21}+g_{m3}R_{1}\beta_{+11}\beta_{+22}}{1+g_{m3}R_{1}\beta_{-11}\beta_{+22}}$$

The non-ideality of transconductance $\;g_{mn}$ can be neglected by satisfying the following condition:

(30) $$\frac{C_{2}g_{m1}\mu_{1}\beta_{-21}\left(B\right)-g_{m1}g_{m2}\mu_{1}\mu_{2}\beta_{-31}\left(C\right)}{C_{1}C_{2}}\ll 1$$

(31) $$\frac{\left(g_{m1}g_{m2}\beta_{-31}\mu_{1}+g_{m1}g_{m2}\beta_{-31}\mu_{2}\right)\left(C\right)}{C_{2}g_{m1}\beta_{-21}\left(B\right)}\ll 1$$

The non-ideal transfer function of the second proposed shadow filter in Figure 4b can be expressed by

(32) $$\frac{V_{LP}1}{V_{in}}=\frac{-V_{LP2}}{V_{in}}=\frac{g_{mn1}g_{mn2}\beta_{-21}\beta_{-22}}{\begin{array}{c} s^{2}C_{1}C_{2}+sC_{2}g_{mn1}\left(\beta_{-21}-g_{mn3}R_{1}\beta_{+31}\beta_{+22}\right)\\ +g_{mn1}g_{mn2}\beta_{-21}\left(\beta_{+21}-g_{mn4}R_{1}\beta_{-41}\beta_{+22}\right) \end{array}}$$

(33) $$\frac{-V_{HP}}{V_{in}}=\frac{s^{2}C_{1}C_{2}\beta_{-22}}{\begin{array}{c} s^{2}C_{1}C_{2}+sC_{2}g_{mn1}\left(\beta_{-21}-g_{mn3}R_{1}\beta_{+31}\beta_{+22}\right)\\ +g_{mn1}g_{mn2}\beta_{-21}\left(\beta_{+21}-g_{mn4}R_{1}\beta_{-41}\beta_{+22}\right) \end{array}}$$

(34) $$\frac{V_{BP2}}{V_{in}}=\frac{-V_{BP1}}{V_{in}}=\frac{sC_{2}g_{mn1}\beta_{-22}}{\begin{array}{c} s^{2}C_{1}C_{2}+sC_{2}g_{mn1}\left(\beta_{-21}-g_{mn3}R_{1}\beta_{+31}\beta_{+22}\right)\\ +g_{mn1}g_{mn2}\beta_{-21}\left(\beta_{+21}-g_{mn4}R_{1}\beta_{-41}\beta_{+22}\right) \end{array}}$$

where $g_{mn3}R_{1}=A_{1}$ and $g_{mn4}R_{1}=A_{2}$

(35) $$\omega_{o}=\sqrt{\left(\beta_{+21}-g_{mn4}R_{1}\beta_{-41}\beta_{+22}\right)\frac{g_{mn1}g_{mn2}\beta_{-21}}{C_{1}C_{2}}}$$

(36) $$Q=\frac{\sqrt{\beta_{+21}-g_{mn4}R_{1}\beta_{-41}\beta_{+22}}}{\beta_{-21}-g_{mn3}R_{1}\beta_{-31}\beta_{+22}}\sqrt{\frac{C_{1}g_{mn2}\beta_{-21}}{C_{2}g_{mn1}}}$$

Using (23), $D\left(s\right)$ of the transfer functions can be rewritten as

(37) $$\begin{array}{l} s^{2}C_{1}C_{2}\left(1-\frac{C_{2}g_{m1}\mu_{1}\left(D\right)-g_{m1}g_{m2}\beta_{-21}\mu_{1}\mu_{2}\left(E\right)}{C_{1}C_{2}}\right)\\ \;\;\;\;\;\;+sC_{2}g_{m1}\left(D\right)\left(1-\frac{g_{m1}g_{m2}\beta_{-21}\mu_{1}\left(E\right)+g_{m1}g_{m2}\beta_{-21}\mu_{2}\left(E\right)}{C_{2}g_{m1}\left(D\right)}\right)\\ \;\;\;\;\;\;+g_{m1}g_{m2}\beta_{-21}\left(E\right) \end{array}$$

where $D=\beta_{-21}-g_{mn3}R_{1}\beta_{+31}\beta_{+22}$, and $E=\beta_{+21}-g_{mn4}R_{1}\beta_{-41}\beta_{+22}$. The non-ideality of the transconductance $g_{mn}$ can be neglected by satisfying the following condition:

(38) $$\frac{C_{2}g_{m1}\mu_{1}\left(D\right)-g_{m1}g_{m2}\beta_{-21}\mu_{1}\mu_{2}\left(E\right)}{C_{1}C_{2}}\ll 1$$

(39) $$\frac{g_{m1}g_{m2}\beta_{-21}\mu_{1}\left(E\right)+g_{m1}g_{m2}\beta_{-21}\mu_{2}\left(E\right)}{C_{2}g_{m1}\left(D\right)}\ll 1$$

Considering the parasitic parameters of DDTA by letting *y*-terminals possess very high impedance levels, which can be neglected, low parasitic resistance $R_{w}$ at *w*-terminal and parallel of parasitic capacitance $C_{o}$ and resistance $R_{o}$ at *o*-terminal. From Figure 3b and Figure 4b, the parasitic parameters $C_{o1}$ and $R_{o1}$ of DDTA_1_ are parallel with $C_{1}$, parasitic parameters $C_{o2}$ and $R_{o2}$ of DDTA_2_ are parallel with $C_{2}$, and parasitic parameters $C_{o3}$ and $R_{o3}$ of DDTA_2_ are parallel with $R_{1}$. These parasitic parameters can be neglected by choosing appropriately values such as $g_{mj}\gg 1/R_{oj}$, $C_{j}\gg C_{oj}$, and $R_{1}\ll R_{oj}$, where j=1, 2, 3 of ${\mathrm{DDTA}}_{j}$.

## 3. Simulation Results

The proposed shadow filters were simulated using SPICE. The MI-DDTA as shown in Figure 1a was designed using a 0.18 μm CMOS technology and the transistor aspect ratios are shown in Table 1. The power supply was ±0.5 V.

**Figure 5:**
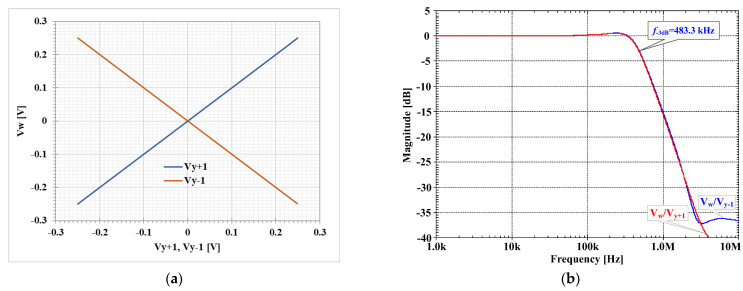
(**a**) DC transfer characteristic $V_{w}$ against $V_{y+1}$ and $V_{y-1}$, (**b**) AC transfer characteristic and −3 dB bandwidth of $V_{w}/V_{y+1}$ and $V_{w}/V_{y-1}$.

Figure 5a shows the DC transfer characteristic $V_{w}$ against $V_{y+1}$ and $V_{y-1}$ of the MI-DDTA while (b) shows the AC transfer characteristic and −3 dB bandwidth of *V_w_/V_y_*_+1_ and *V_w_/V_y_*_−1_ with load capacitance of 10 pF, the −3 dB bandwidth is around 483.3 kHz and the low frequency gain is −0.016 dB. It is notable the capability of operation in a wide range of the input voltages. Note, that both, the differential, as well as common-mode range of the input differential amplifier M1–M2 is increased 1/*α* times, thanks to the input capacitive divider.

**Figure 6:**
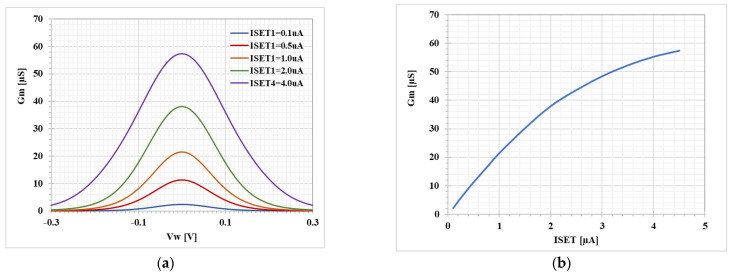
Transconductance $G_{m}$ with different values of $I_{SET}$: (**a**) $G_{m}-V_{w}$, (**b**) $G_{m}-I_{SET}$.

Figure 6 shows the small-signal transconductance of the transconductance stage, against the input voltage $V_{w}$_,_ for different $I_{\mathrm{SET}}$ (Figure 6a), and against I_SET_ (Figure 6b). It is worth noting that although the input range is sufficient for the proposed applications, this range, if needed, could be simply increased using a linearization technique like the source degeneration that results in increased dynamic rang of the system. The parasitic parameters of DDTA are *R_y_* = 6.28 GΩ, $R_{w}$ = 540 Ω, $R_{o}$ = 11.9 MΩ, and $C_{o}$ = 33.67 fF.

The first proposed shadow filter was designed with C_1_ = C_2_ = 3.3 nF and R_1_ = 46.5 kΩ. Note that these passive values are sufficient to avoid the impact of parasitic effects. The transconductance $g_{m3}$ adjusted by the current I_SET3_ was used to control the amplifier A. The bias currents I_SET1_ and I_SET2_ were used to control $g_{m1}$ and $g_{m2}$, respectively.

The first simulation was performed with A = 0, by setting the bias current I_SET3_ = 0 and I_SET1_ = I_SET2_ = 1 μA ($g_{m}=$ 21.5 μS). This setting resulted in natural frequency (*f*_o_) of 1.036 kHz and the quality factor (*Q*) of 1.

**Figure 7:**
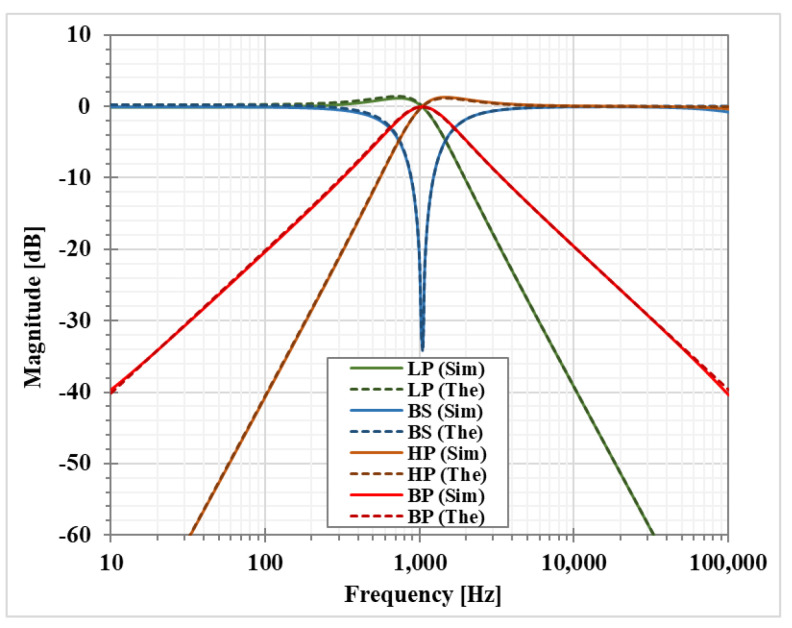
Simulated LP, HP, BP and BS magnitude frequency responses of the first shadow filter without modification of the natural frequency and the quality factor (Sim = Simulation, The = Theoretical).

**Figure 8:**
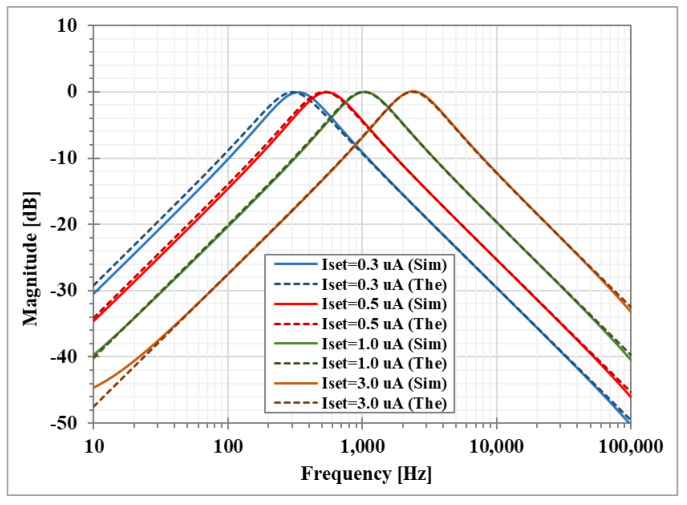
Simulated BP magnitude frequency responses of the first shadow filter with modification of the natural frequency via the bias currents (I_set_ = I_set1_ = I_set2_).

**Figure 9:**
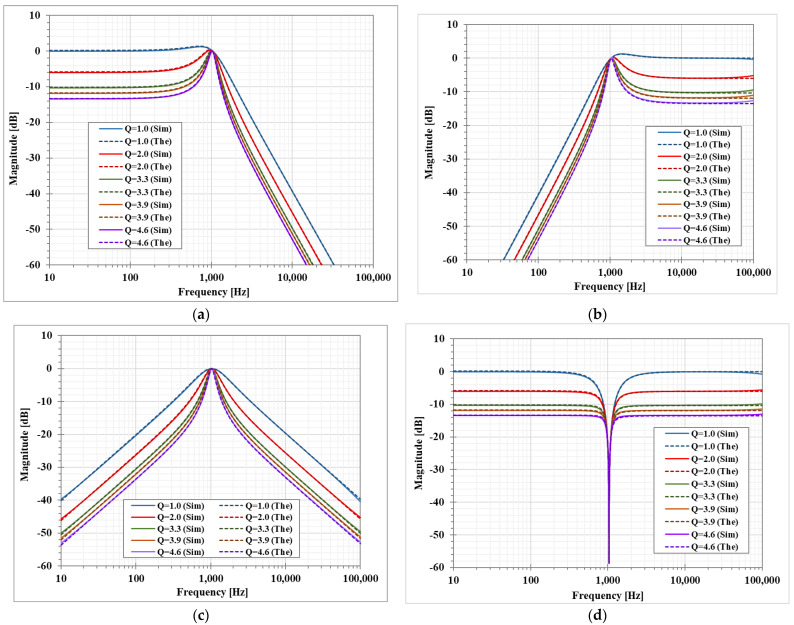
Simulated magnitude frequency responses of the first shadow filter with setting the quality factor by the amplifier A (**a**) LP, (**b**) HP, (**c**) BP, and (**d**) BS.

The magnitude responses of the LP, HP, BP, and BS filters are shown in Figure 7. Figure 8 shows the magnitude responses of the BP filter when the bias currents I_SET1_ = I_SET2_ are varied. This result confirms that the natural frequency of the shadow filter can be electronically controlled. Figure 9 shows the magnitude frequency responses when the amplifier A is used to set the quality factor *Q* equal to 1.0, 2.0, 3.2, 3.9, and 4.6. Figure 9c shows that the quality factor of the BP filter can be controlled by the amplifier A (A > 1) with the passband gain equal to 0 dB while the passband gain of the LP, HP and BS filters in Figure 9a–c respectively, will decrease when the quality factor is increased.

**Figure 10:**
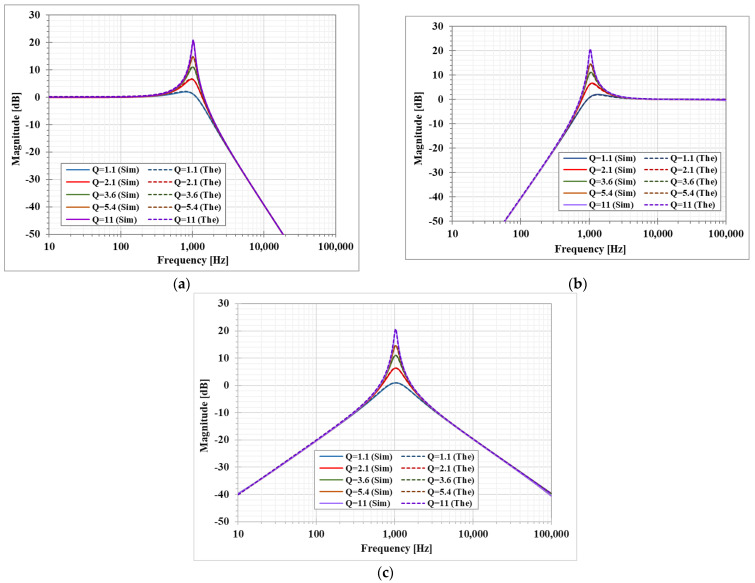
Simulated magnitude frequency responses of the second shadow filter with setting the quality factor by the amplifier A_1_, with A_2_ = 0 (**a**) LP, (**b**) HP, and (**c**) BP.

The second proposed shadow filter was designed with C_1_ = C_2_ = 3.3 nF and R_1_ = 46.5 kΩ. The bias currents I_SET1_ and I_SET2_ were used to adjust $g_{m1}$ and $g_{m2}$, respectively. The $g_{m3}$ that was adjusted by I_SET3_ and $g_{m4}$ adjusted by I_SET4_ were used to control the amplifiers $A_{1}$ and $A_{2}$, respectively. The first simulation was performed with $A_{2}$ = 0 by setting the bias currents I_SET4_= 0, I_SET1_ = I_SET2_ = 1 μA ($g_{m}=$ 21.5 μS), while the amplifier $A_{1}$ was used to control the parameter *Q*. The simulated magnitude responses of the LP, HP, and BP filters with *Q* = 1.1, 2.1, 3.6, 4.5, 10.0 are shown in Figure 10. This result confirmed that the parameter *Q* can be controlled by $A_{1}$, without affecting the parameter $\omega_{o}$.

**Figure 11:**
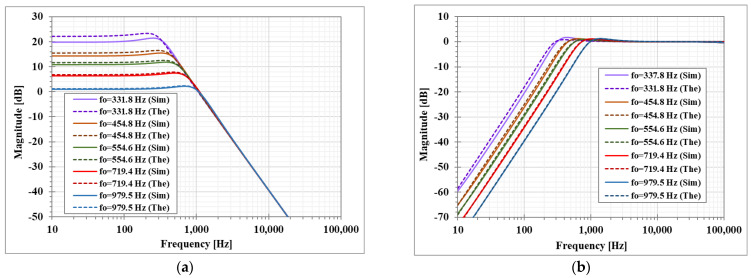

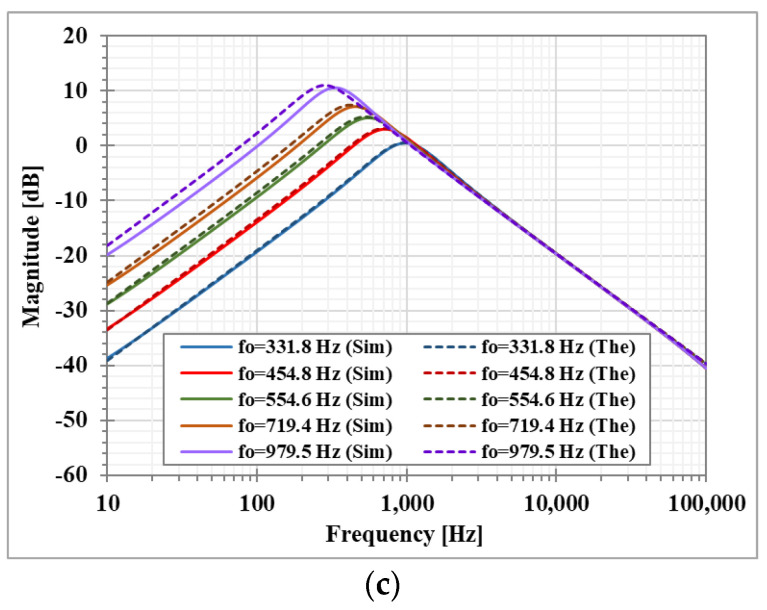
Simulated magnitude frequency responses of the second shadow filter with setting the natural frequency by A_2_, while A_1_ is used to adjust Q=1, (**a**) LP filter, (**b**) HP filter, (**c**) BP filter.

Figure 11 shows the simulated magnitude frequency responses of the LP, HP, and BP filters when the amplifier $A_{2}$ gain was regulated by $g_{m4}$ and the amplifier $A_{1}$ was used to control the parameter *Q* = 1. This result confirmed that when the parameter $\omega_{o}$ is varied by the amplifier $A_{2}$, the passband gain of the LP and BP filter is changing while the passband gain of the HP filter is constant.

**Figure 12:**
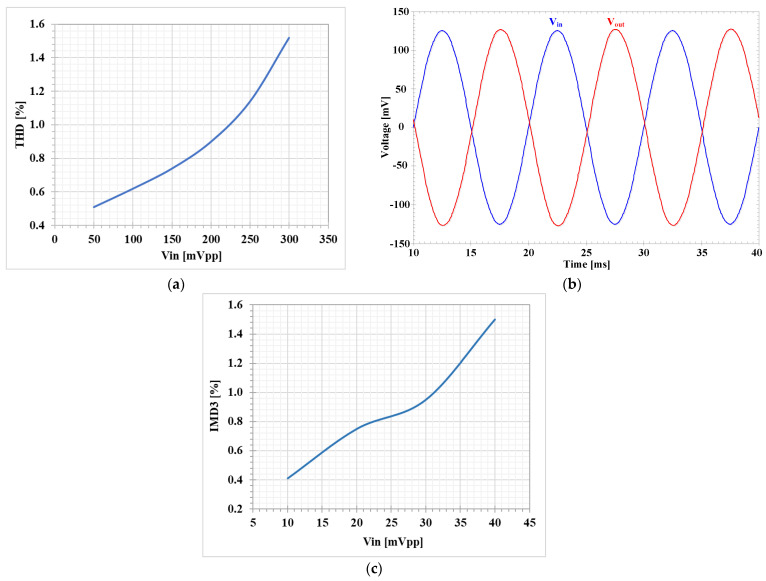
Simulated distortion of first proposed shadow filter: (**a**) THD of the LP filter, (**b**) the transient response of the LPF with THD lesser than 1.2 % for V_in_ =250 mV_pp_, and (**c**) IMD3 of the BP filter.

Figure 12a shows the simulated total harmonic distortion (THD) of the LP filter for *f* = 100 Hz. The amplifiers A_1_ and A_2_ were not active (I_SET3_ = I_SET4_ = 0 A). It can be noticed that THD was less than 1.2% for V_in_ < 250 mV_pp_ and its transient response is shown in Figure 12b. Figure 12c shows the simulated third intermodulation distortion (IMD_3_) of the BP filter for a two-tone test, with two closely spaced tones of *f*_1_ = 0.9 kHz and *f*_2_ = 1.1 kHz. The IMD_3_ was less than 1.5% for the input amplitude up to 40 mV_pp_.

**Figure 13:**
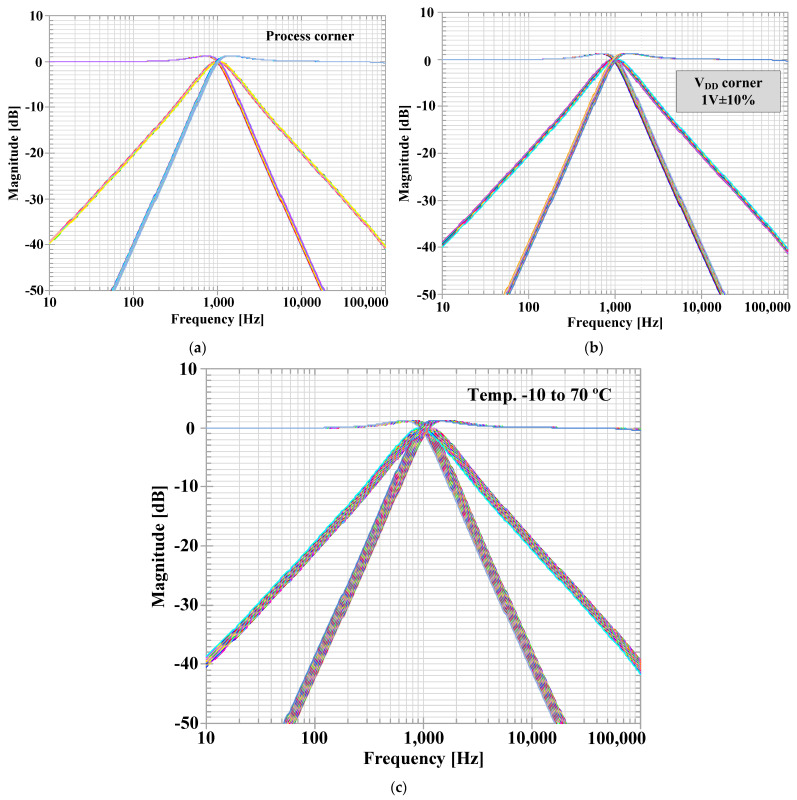
Simulated magnitude responses of the first proposed shadow filter: (**a**) process corner, (**b**) voltage corner, and (**c**) temperature.

The simulated magnitude responses of the LP, HP, and BP filters for process, voltage, and temperature (PVT) corners were investigated. Figure 13a–c show respectively the results of Monte Carlo (MC) analysis, were variations of the threshold voltages of MOS transistors by 10% (LOT tolerance), supply voltages by +/− 10% and temperature from −10 °C to 70 °C were assumed. As it can be noticed, the proposed filter is robust under the assumed PVT variations.

Finally, Table 2 provides a comparison of the proposed filters with previously published shadow filters in [22,23,26,29,30]. The proposed filters provide lower power consumption, as compared with [22,23], lower output impedance, as compared with [26,30] (except the output impedance of the LP filter in Figure 3b), larger number of low-impedance nodes, as compared with [29], and lower supply voltage, as compared with [23,26,29,30].

## 4. Conclusions

This paper presents new voltage-mode shadow filters with single-input multiple-output topology, using low-voltage low-power multiple-input differential difference transconductance amplifiers. The multiple-input DDTA can be easily realized using MIGD-MOST technique. The proposed filters offer high-input impedance and most of the output terminals offer low-impedance. The natural frequency and the quality factor of the filters can be electronically and independently controlled. The impact of the non-idealities of the DDTA on the performance of the proposed shadow filter is studied. The SPICE simulation results using 0.18 μm CMOS process from TSMC is given to validate the workability of the new circuits.

## Figures and Tables

**Table 1:** Parameters of the components of MI-DDTA.

Transistor	W/L (µm/µm)
DDA	
M_1_, M_2_	90/3
M_3_	180/3
M_B_, M_4_, M_5_	30/3
M_6_	60/3
M_7_	150/3
M_L_ (R_MOS_)	4/5
C_G_ = 0.5 pF, C_C_ = C_B_ = 2.6 pF	
I_B_ = 1 μA	
TA	
M_8_, M_9_, M_15_, M_16_, M_B1_, M_14c_	30/1
M_10_, M_11_, M_12_, M_13_	20/1
M_10c_, M_11c_, M_12c_, M_13c_	10/1
M_15c_, M_16c_, M_B1c_	15/1
M_14_	60/1

**Table 2:** Comparison of the Proposed Shadow Filters with Previous Works.

Parameters	[22] Figure 4b	[23]	[26]	[29]	This Work Figure 3b	This Work Figure 4b
Technology [μm]	0.35	0.18	0.18	0.18	0.18	0.18
Supply voltage [V]	±0.5	±0.9	±1.5	±0.9	±0.5	±0.5
No. of ABB	4-DDCC	1-VDTA	4-VDTA	3-VDDA	3-DDTA	4-DDTA
No. of R & C	5 + 2	0 + 2	0 + 2	1 + 2	1 + 2	1 + 2
High input impedance	Yes	Yes	Yes	Yes	Yes	Yes
Low output impedance	No	No	No	HP, AP	HP, BP, BS	Yes
Availability of responses	LP, HP, BP	LP, BP	LP, BP	LP, HP, BP, BS, AP	LP, HP, BP, BS	LP, HP, BP
Electronic control of $\omega_{o}$ and Q	No	Yes	Yes	Yes	Yes	Yes
Power consumption [μW]	184	3620	-	-	24.9	30
THD [%]	-	-	-	1@200 mVpp	1.14@250 mVpp	
IRN [$\mu V/\sqrt{Hz}]$	-	-	-		62.6	
Dynamic rang [dB]	-	-	-		62.9	
Verification of result	Sim./Exp.	Sim.	Sim.	Sim./Exp.	Sim.	
